# Flexible ureteroscopy update: indications, instrumentation and technical advances

**DOI:** 10.4103/0970-1591.44263

**Published:** 2008

**Authors:** Srinivas Rajamahanty, Michael Grasso

**Affiliations:** St Vincent's Medical Center Campus, New York Medical College, New York, USA

**Keywords:** Instruments, flexible ureteroscope, accessories

## Abstract

Retrograde ureteroscopy has recently gained a broadened indication for use from diagnostic to a variety of complex minimally invasive therapies. This review aims to look at the recent advances in the instrumentation and accessories, the widened indications of its use, surgical techniques and complications. With minimization of ureteroscopic instruments manufacturers are challenged to develop new, smaller and sturdier instruments that all will also survive the rigors of surgical therapy.

## INTRODUCTION

Ureteroscopy is defined as retrograde instrumentation performed with an endoscope passed through the lower urinary tract directly into the ureter and calyceal system.[1] With the addition of actively deflectable, flexible endoscopes the indications for ureteroscopy have broadened from diagnostic to a variety of complex minimally invasive therapies. Current ureteroscopic treatments include intracorporeal lithotripsy (by far the most common), treatment of upper urinary tract urothelial malignancies, incising strictures, evaluation of ureteral trauma, and repairing ureteropelvic junction obstructions.[2,3] With improved instrumentation and incorporation of technologies such as a large endoscope working channel and active tip deflection, the evolution of surgical techniques have broadened while the complications noted with ureteropyeloscopy have actually decreased significantly.[4,5]

## MECHANICS

The application of flexible ureteroscopy was first reported by Marshall in 1964. A 9F fiberscope manufactured by American Cystoscope Makers (Pelham Manor, NY) was passed into the ureter to visualize an impacted ureteral calculus. Subsequently, Bagley, Huffman, and Lyon began work at the University of Chicago to develop an improved flexible fiberoptic ureteropyeloscope in the 1980s.

**Figure 1:**
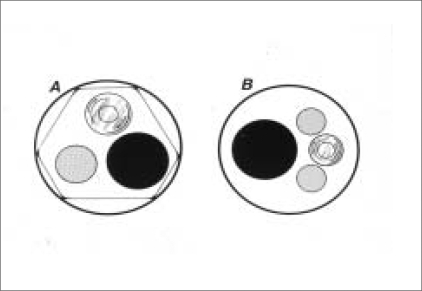
Cross-section view of flexible ureteroscope showing optical system, working channel and deflection mechanism

Three major design changes improved the therapeutic potential of the flexible ureteroscope [Figure 1].

The optical system consists of fiberoptic light bundles created from molten glass. Each glass fiber is cladded with a second layer of glass of different refractive index to improve the internal reflection, light transmission and also the durability of the endoscope. When the fibers are bundled randomly, they provide excellent light transmission for illumination, but no image. However, if the fibers are placed in a coherent fashion, the light from each fiber will coalesce to transmit images. Small lenses placed proximally and distally enable a telescopic effect with image magnification, increased field of view and focusing ability. A recent modification is the splitting of the light bundle distally to enable a more central placed working channel and better distribution of light within the working field of view.[5]

The deflection mechanism of the flexible ureteroscope permits maneuverability within the collecting system of the kidney. This deflection is usually provided by several wires running down the length of the endoscope and attached to a lever which is manually operated. Manipulating the lever will deflect the tip. If the tip moves in the same direction of the lever, the defection is described as “intuitive”- i.e. down is down and up is up. In the past, prior to 1992, deflection was active at the tip and secondary deflection along the shaft was passive. To obtain lower pole access, the urologist would maximally deflect and advance the tip of the endoscope.[6] The secondary deflection was achieved by the endoscope passively buckling at a set designed point along the shaft. In 1992, Karl Storz (KSEA, Tuttilegan, Germany) was able to downsize the flexible endoscope from 9.8 Fr to 7.5 Fr while maintaining the same 3.6 Fr working channel. This milestone event allowed all urologists to more easily pass the endoscope and in so doing broaden the therapeutic applications. The current instruments have continuous controlled dual deflection with increased downward and upward deflection up to 270 degrees, referred to as “exaggerated deflection” in both directions. This deflection is performed with a single more ergonomic lever as compared to the cumbersome two separate levers employed by the ACMI DUR 8 (Gyrus Inc, London, England). The radius of deflection is also broader, thereby enabling more maneuverability and permitting placement of instruments in the lower pole. The most modern endoscopes also incorporate a shock absorbing system (a form of secondary deflection) which is located proximal to the active deflecting system and allows for gentle rolling of the distal end for approximately ten centimeters enabling access more deeply into the calyces.[7]

The working channel permits placement under direct vision of a variety of accessories including graspers, baskets, wires and laser fibers through the endoscope. All current endoscopes have a channel of at least 3.6 Fr which allows the use of instruments up to 3 Fr while still permitting concurrent irrigation. The composition material of the accessory influences tip deflection. For example, graspers and baskets with a shaft composed of polyamide tend to be stiffer and inhibit deflection as compared to Teflon sheathed accessories.[8]

Many ureteroscope repairs are due to damage to the working channel from malfunction or incorrect use of the holmium laser. This is often a technical issue when the fiber firing end is located too close to the endoscope tip. The new-generation Storz endoscopes incorporate a bead-like sequence of hollow ceramic rings in the distal end of the working channel for 1.5 cm. This protects the instrument from thermal or electrocautery damage and allows the endourologist to work closer to the tumor, stricture or stone while using laser energy.

## DURABILITY

White and Moran reported the need for major urteroscope repairs after only 12 endoscope usages.[9] Afane et al., demonstrated that flexible ureteroscopes from four major manufacturers required major repairs after only 15 procedures or 13 h of usage.[10] Traxer *et al.*, from Paris performed 50 flexible ureterosopies using the Karl Storz Flex-X ureteroscope. They evaluated the maximal active ventral and dorsal deflection, irrigation flow at 100 cm H20 and number of broken optical fibers. The maximal ventral deflection deteriorated from 270 degrees initially to 208 following the last procedure; the maximal dorsal deflection decreased from 270 to 133 degrees. The irrigation flow at 100 cm H20 decreased from 50 to 40 after the last procedure. They concluded that the need for repair occurred less frequently with the newer generation endoscopes and when used by an experienced endourologist.[11] In general most centers can employ these instruments for approximately 50 cases between repairs with damage and breakage occurring most often during sterilization.

Irrigating fluids are employed to clear the optical field of view and to cool the tip of energy-delivering devices. The irrigant is delivered through the same channel used for working instruments, often through a side arm adapter (Urolock – Boston Scientific, Natick Mass. and Check flow, Cook Urologic, Spencer, Indiana). The simplest and most cost-effective means of delivering continuous irrigant is to employ two 60 cc syringes connected to a three-way stopcock with arterial line tubing. Normal saline is the irrigation standard solution for diagnostic ureteroscopy and lithotripsy. When electrocautery is employed sorbitol or small aliquots of sterile water may be used.[12]

## ACCESSORIES

Accessories include guide wires, stone retrieval devices, access sheaths, electrodes, laser fibers, biopsy forceps, etc. With regard to guide wires, the PTFE -coated stainless steel guide wire and the Zebra wire (PTFE coated with nitinol core – Boston Scientific, Natick Mass) are useful to help facilitate endoscope tip access to the ureter in routine cases. The Terumo Glide wire is particularly useful in cases of difficult ureteral access. It is employed as an access guide wire and not a working guide wire. This means that the very lubricious coating is useful in bypassing an obstruction, and can facilitate ureteral catheter placement, while the slippery nature of this nitinol design frequently does not aid in placing the larger endoscope.

Several new unique guide wires are now available including the Sensor wire (Boston Scientific, Natick, Mass.). The Sensor guide wire, for example, has a smooth hydrophilic nitinol-based distal tip, a kink-resistant body made of nitinol alloy core, and PTFE-coated stainless steel jacket which adds stiffness and helps prevent endoscope buckling during endoscope placement into the ureter. This guide wire also has a flexible proximal tip for atraumatically back loading the wire through the working channel of the ureteroscope.[13,14]

The ureteral access sheaths can facilitate repeat ureteroscopic access to ureter. These sheaths range from 12-14 Fr and enable repeated passage of the ureteroscope without a guide wire. The advantages include easy endoscope placement and possible decreased intrarenal irrigant pressures. The disadvantages include over-dilation for placement, false sense of security, and potentially increased rate of ureteral stricture from prolonged use.[15]

## FUTURE ADVANCES: THE DIGITAL FLEXIBLE URETEROSCOPES

Recently, flexible digital ureteroscopes have been introduced. These endoscopes have an integrated light source and distal digital chip-based camera. The distal chip camera system requires a larger outer endoscope diameter which is an issue for access, while the image quality is equivalent to ten times the pixel resolution of standard fiber optic endoscopes. Since these instruments do not require a separate camera head or light cord, they may potentially be more durable.

More studies are needed before concluding that these more costly additions are superior to the conventional fiber optic flexible endoscopes. Early issues include digital processing of colored light, especially red light, and problems with chip stability during laser lithotripsy where the created acoustic percussions distort the digital images.

## TABLE 1: INDICATIONS FOR URETEROSCOPY[16,17]

**Table 1:** Current Indications for therapeutic flexible ureteroscopy

Endoscopic Lithotripsy	186	40%
Upper Pole	34	
Middle Pole	38	
Lower Pole	66	
Renal Pelvic	23	
Ureter	56	
Stienstrasse	3	
Treat Upper Tract TCCa	119	26%
Diagnostic	107	23%
Incise Ureteral Stricture	21	5%
Assisted PCNL	8	2%
Retrograde Endopyelotomy	5	2%
Caliceal Diverticulum	4	1%
Complex Infundibulotomy	4	1%
Foreign Body Removal	2	
Treatment of Hemangioma	3	
Submucosal Stone	1	
Internal Drainage Parapelvic Cyst	1	
Total	460	

(Advanced Ureteroscopy: Wireless and Sheathless. J Endourol. 2006)[20]

### Diagnostic indications

Abnormal imaging findings - Filling defect
Obstruction - Determination of etiology
Unilateral essential hematuria
Localizing source of positive urinary cytology results, culture results, or other test results
Evaluation of Ureteral injury

### Therapeutic indications

Endoscopic lithotripsy
Retrograde endopyelotomy
Incision of ureteral strictures
Improvement of calyceal drainage
Treatment of calyceal diverticular lesions
Treatment of malignant urothelial tumors
Treatment of benign tumors and bleeding lesions
Treatment of Retained Stents

## SURGICAL TECHNIQUE

### Ureteral access

The intramural ureter is the narrowest segment and can prohibit endoscope passage. Guide wires often are passed into the ureteral orifice cystoscopically and then directed into the renal pelvis with fluoroscopic assistance. These “safety” guide wires straighten the ureter and facilitate both the dilation of obstructed segments with balloon or graduated dilators and the placement of internal stents.[17]

Historically, the intramural ureter required dilation for endoscope access. Currently, the small-diameter flexible ureteroscopes often have less than 7.5 F tip diameter, and can be passed without any formal dilation. Use of a dilator to facilitate passage of the ureteroscope beyond the intramural tunnel is recommended when the ureter is narrow or restrictive. This is common in the young male population. Otherwise, the use of such dilators or operative sheaths is optional and generally not required.[18]

One access method is to employ a 10 Fr dual lumen catheter first over the initial access guide wire. This aids in both dilating the intramural ureter and in facilitating passage of a second “working” guide wire. This scheme is useful when the ureter is tortuous or J-hooked distally. The orifice can also be dilated with a balloon dilator (most commonly 12 F for access) and a second working wire passed beside. The flexible ureteroscope is next passed over the working guide wire in a monorail fashion into the ureter and the working guide is removed. Alternatively, the smallest diameter ureteroscopes (7.5 F tipped) can be passed directly into the ureter under direct vision without guide wire assistance. Fluid irrigation facilitates flexible ureteroscope optical visibility. Although automatic pumps are available for this purpose, hand irrigation is often preferred.[19]

In a recent prospective study of 460 consecutive upper-tract endoscopies at our center, “no-touch” direct access ureteroscopy (i.e. placement of the endoscope into the ureter under direct vision without the assistance of a guide wire and without dilation) was successfully performed in the majority of patients. This wireless form of flexible ureteroscopy or “no touch technique” is technically challenging but eliminates the potential trauma, mucosal irritation and inadvertent manipulation of stones or tumors caused by guide wires and is particularly helpful when mapping the collecting system for mucosal lesions or upper tract transitional cell cancers.[20]

Another access technique is to pass the tip of a guide wire through the endoscope just beyond any blockage, or kink in the ureter and then follow with the ureteroscope until it rests beyond the obstruction. This will open or straighten ureteral segments, often allowing easier passage.

Lower-pole intrarenal access performed with a flexible ureteroscope is often challenging and commonly requires both active and passive flexible ureteroscope deflections. To place the tip of the endoscope into the lower pole, the instrument must first be actively deflected and then advanced so as to allow the shaft below to buckle. This maneuver, termed secondary deflection, is required in 60% of traditional flexible ureteroscopies if a complete inspection is to be attained. The increased active tip deflection offered by new-generation flexible ureteroscopes significantly decreases the need for secondary deflection and enhances the surgeon's ability to inspect all aspects of the renal collecting system. Fluoroscopic guidance is frequently employed to provide a road map of the intrarenal collecting system. The flexible ureteroscope is directed from calyx to calyx, and frequently dilute contrast material is injected through the working channel of the endoscope to help ensure the entire collecting system is mapped.[21,22]

If electrocautery is to be employed, special attention to the guide wire choice helps prevent intraoperative complications. If a standard stainless steel guide wire is used, electrical current may inadvertently arc to the wire during cautery use and cause excessive ureteral coagulation with subsequent fibrosis and stricture formation. This can be prevented by using an insulated guide wire such as a Teflon-sheathed nitinol Zebra wire (Boston Scientific, Natick, Mass.).

### Endoscopic lithotripsy

Endoscopic lithotripsy is the most common indication for flexible ureteroscopy. Holmium laser energy is by far the most efficient endoscopic lithotrite. The smallest diameter laser fiber (200 micron) helps facilitate complete tip deflection and is useful when lower pole stones are being addressed. The vaporization bubble created when this laser energy is delivered in a water-based irrigant increases exponentially with larger fiber diameters. The 365 micron fiber, for example, more efficiently clears stone, but the stiffer nature of this fiber limits its use in the intrarenal calyceal system.[23]

There are a variety of schemes employed to treat intrarenal calyceal stones with the flexible ureteroscope and laser lithotripter. One common technique is to manually move stones from a dependent calyx with a flexible three-prong grasper or nitinol-based loop extractor like the Graspit. Once repositioned, the stones can be more efficiently fragmented with a larger diameter laser fiber. For the largest stones the most efficient technique is to core out the central component, creating mostly dust, and then reduce the periphery to small fragments 1 to 2 mm in diameter which are left to pass over time beside a ureteral stent. For smaller stones, however, multiple endoscope passes to extract fragments, with or without a sheath, may be performed.

Internal ureteral stents are associated with lower urinary tract symptomatology, which includes urinary frequency, urgency, and mild-to-moderate hematuria, which is transient. If ureteral dilation is required for ureteroscope access, and/or the endoscopic procedure is complex (large stone burden, ureteral tortuosity, etc.) a ureteral stent is placed at the end of the endoscopic procedure to ensure drainage. Ureteral stents are also useful in that they can help straighten a tortuous ureter and facilitate passive dilation which is useful in clearing stone fragments. Removal of ureteral stents is performed after a period of healing that can range from a few days to six to eight weeks, depending on the complexity of the treatment. Stents are removed most commonly in the office with either an attached nylon tether or cystoscopically.[24]

Most ureteroscopic lithotripsies are performed as day surgery outpatient procedures. Patients are discharged on prophylactic oral antibiotics and analgesics. Anticholinergic medications and alpha-blockers may be used to minimize symptoms of frequency, urgency, and discomfort often associated with ureteral stents; however, individual patient tolerance varies. Choosing the correct stent length (based on the ureteral length) and optimal positioning help to minimize unpleasant symptoms.

## COMPLICATIONS

### Minor intraoperative complications

In general, the minor complication rate from ureteropyeloscopy has decreased based on refined technique, experience of the operators, and prompt treatment or prevention of intraoperative problems. Prophylactic parenteral antibiotics, careful guide wire placement, minimization of excessive ureteral dilation, and postoperative ureteral stenting all impacted on the rate of postoperative problems. This, combined with better surgical training and improved instrumentation, resulted in this very positive trend.

### Major intraoperative complications

The major complication rate associated with therapeutic ureteroscopy has decreased markedly and currently occurs in less than 1% of all procedures. As with the minor problems, major complications occur less frequently for basically the same reasons – better surgeon skills and improved instrumentation. However, when they do occur treatment is often more complex. In addition to major intraoperative problems, other complications that occur during upper urinary tract endoscopy may begin as minor events and, if left untreated or if addressed incorrectly, can progress to more serious conditions.

Major ureteral wall perforations occur infrequently and can be the product of a heavy-handed endoscopist and improper application of the ureteroscope. These complications are more common with the semi-rigid ureteroscopes rather than the flexible ureteroscopes. The forceful positioning of a semi-rigid ureteroscope above the iliac vessels, particularly in young male patients, is associated with a significant risk of ureteral wall trauma unless the collecting system is dilated or the ureter has been stented prior to endoscopy. Routine use of a double-J stent is not necessary in most patients but is recommended when unusual difficulty is encountered or when extensive strictures are noted. It is essential to note that if the endoscopic maneuvers are difficult, the surgeon can only be rewarded with an easier time in the future if he does not push the procedure but rather places a stent and returns another day. Usually, one to two weeks of stenting greatly facilitates ureteroscopy, particularly if proximal access is desired.

Care must be taken when treating stones in the ureter. Ureteral wall perforation with stone migration into the defect can lead to formation of a stone granuloma and/or ureteral wall stricture. In addition, attempts at extracting a particularly large stone with a basket rather than fragmenting it can lead to a ureteral perforation or avulsion. The general rule is if a stone or fragment is too large to pass on its own, trying to extract it with an accessory without reducing its size with an endoscope lithotrite has inherent risk.[25]

When distal ureteral avulsion is noted, ureteroneocystostomy repair can be performed, with a psoas bladder hitch if necessary to create a tension-free anastomosis. A Boari bladder wall flap will increase the proximal extent of the repair to the middle third of the ureter. These repairs are performed most commonly over a ureteral catheter with perianastomotic drainage. This can be performed acutely at the time of the injury or in a staged fashion after proximal percutaneous drainage is obtained at the time of the injury.

If the entire devitalized ureteral segment is inadvertently brought into the bladder, it is of no value in subsequent repair. Percutaneous renal drainage should be obtained immediately at the time of this type of ureteral injury. Subsequent therapy is based on either bowel interposition (i.e., ileal ureter) or renal auto transplantation to a pelvic position. Both procedures are complex and should be performed in a staged fashion after a period of healing Table 2.

**Table 2:** Comparison of complication rates associated with ureteroscopy, emphasizing the noticeable decrease in the major complication rate with greater experience and endoscope miniaturization[26–29]

	Blute	Abdel Razzak	Harmon	Grasso	Zhang Wu
Year	1988	1992	1997	2001	2007
Patients	346	290	209	1000	1443
Minor Complication				
False Passage	0.9%	-	-	0.4%
Fever	6.2%	6.9%	2.0%	1.3%
UTI	-	1.0%	-	1.7%
Pyelonephritis	-	-	-	1.0%
Major Complications				
Major Perforation	4.6%	1.7%	1.0	0	0.5%
Stricture	1.4%	0.7%	0.5%	0.4%	0.3%
Avulsion	0.6%	0	0	0
Urinoma	0.6%	-	0	0
Urosepsis	0.3%	0	0	0
CVA, DVT, MI	-	-	0.5%	0.3%

## CONCLUSION

Miniaturization of ureteroscopic instrumentation will continue, with smaller fiber optics, improved mechanics facilitating lower pole access, improved accessories, and new energy sources. As the instrumentation becomes smaller and more refined, it also will become more delicate. Thus, manufacturers are challenged to develop new, smaller instruments that will also survive the rigors of surgical therapy.
